# New light on chemotherapy toxicity and its prevention

**DOI:** 10.1038/s44276-024-00064-8

**Published:** 2024-05-22

**Authors:** Ronit Juthani, Sachin Punatar, Indraneel Mittra

**Affiliations:** 1 Translational Research Laboratory, Advanced Centre for Treatment, Research and Education in Cancer, Tata Memorial Centre, Kharghar, Navi Mumbai, 410210 India; 2 Homi Bhabha National Institute, Anushakti Nagar, Mumbai, 400094 India; 3 Department of Medical Oncology, Tata Memorial Centre, Kharghar, Navi Mumbai, 410210 India

## Abstract

Most patients with cancer receive chemotherapy. Unfortunately, chemotherapy is associated with a number of potentially life-threatening side effects. There is a need to ameliorate chemotoxicity to improve therapeutic outcomes and quality of life. Chemotoxicity arises from systemic DNA damage and inflammation in healthy cells due to chemotherapy drugs. Traditionally, these processes are believed to be caused by the direct death of normal cells by chemotherapeutic drugs. However, new research has challenged this dogma by suggesting that chemotoxicity is a secondary effect associated with the release of cell-free chromatin particles (cfChPs) from cells subjected to drug-induced death. Released cfChPs can freely enter into bystander healthy cells to inflict double-strand (dsDNA) breaks and activate inflammatory and apoptotic pathways. The drug-induced cell death and cfChPs release have cascading effects that exaggerate and prolong chemotoxicity. Furthermore, evidence has emerged from laboratory and preclinical studies, and two phase II clinical trials, indicating that chemotoxicity can be minimised by deactivating cfChPs. Three cfChPs-deactivating agents have been identified, of which the nutraceutical combination resveratrol and copper (R–Cu)—easily administered orally and with little toxicity—is the agent of choice for human therapeutic use. This article aims to provide practising medical oncologists with a perspective on this emerging research on chemotoxicity and its prevention and its potential implications for the future. Well-designed randomised clinical trials will be necessary to establish the true clinical value of these findings in day-to-day practice.

## Introduction

Globally, over 19 million new cancer cases are detected annually [1]. Most individuals diagnosed with cancer receive some form of chemotherapy, with or without additional treatment, and experience varying degrees of toxic side effects. Indeed, the clinical spectrum of toxicities is vast, ranging from less severe toxicities, such as nausea, vomiting, dysgeusia, and hair loss, to those that are more serious and potentially life-threatening including myelosuppression, febrile neutropenia, severe oral mucositis, and sepsis [2, 3]. In addition to these acute toxicities, several late effects are associated with chemotherapy, involving the cardiac [4, 5], neurological [6], renal [7], pulmonary [8], and hepatic systems [9]. Estimates indicate that more than a quarter of patients receiving conventional chemotherapy experience severe (grade IV) potentially life-threatening toxicities [10]. Several therapies are prescribed to mitigate the toxic effects of chemotherapy, with variable success rates.

Chemotherapy-induced toxicity results from systemic DNA damage and inflammation in healthy cells [11], traditionally believed to be caused by the direct death of normal cells by chemotherapeutic drugs [12]. However, recent research has challenged this belief by suggesting a novel mechanism of chemotoxicity involving cfChPs that are released from cells subjected to drug-induced death [13–15], with evidence indicating that the toxicity of chemotherapy can be minimised by deactivating cfChPs [16]. This evidence has been sourced from laboratory studies at the cellular level, pre-clinical animal studies, and clinical trials in patients with cancer, each of which is discussed in detail below.

## Laboratory studies

**Fig. 1:**
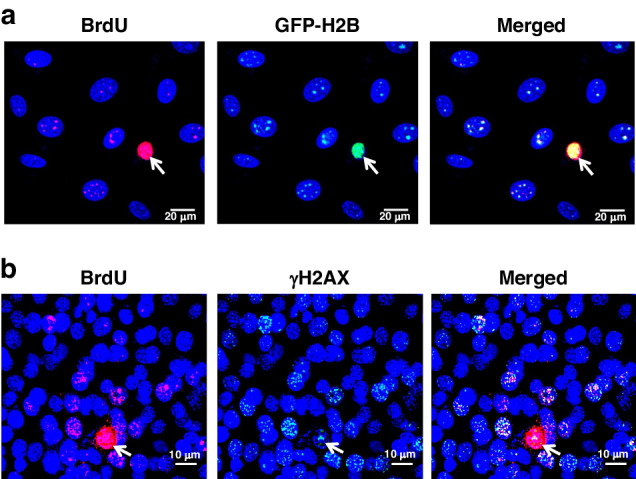
Representative Immunofluorescence images to illustrate chemotherapy-induced bystander effect. **a** MDA-MB-231 human breast cancer cells were dually fluorescently labelled in their DNA with BrdU and in their histones with H2B-GFP according to a method described by us earlier [15] The dually labelled cells were treated with Adriamycin for 48 h and the dead cells were mixed with healthy NIH3T3 mouse fibroblast cells at a ratio of 1:30 and plated on coverslips in a culture dish. After 24 h of incubation, the cells were fixed and examined by fluorescent microscopy. The dead MDA-MB-231 cell is marked with an arrow. Dually labelled cfChPs are clearly detected in bystander NIH3T3 cells. **b** B16F10 mouse melanoma cells were pre-labelled in their DNA with BrdU and killed with Adriamycin. The dying cells were mixed with normal NIH3T3 mouse fibroblast cells at a ratio of 1:30 and plated on coverslips on a petri dish. After 24 h of incubation, the cells were fixed and stained with antibodies against BrdU and γH2AX. BrdU-labelled DNA particles from the dying B16F10 melanoma cell (marked with arrow) are clearly seen in bystander NIH3T3 cells which co-localise with γH2AX signals indicating dsDNA breaks in them.

The mechanism of the bystander effect, which is characterised by damage to non-targeted and often distant cells in association with chemotherapy (and radiotherapy) [16, 17], has perplexed scientists for decades. For example, when Adriamycin-killed lymphocytic leukaemia (Jurkat) cells were co-cultured with normal NIH3T3 cells, the bystander NIH3T3 cells showed upregulation of markers of DNA damage, DNA repair, and apoptosis in the form of increased expression of phosphorylated histone H2AX (γH2AX), phosphorylated ataxia-telangiectasia mutated protein (p-ATM), and caspase-3, respectively; pro-inflammatory markers, including NF-κB, TNF*α*, IFN-*γ*, and IL-6, also showed notable upregulation [14]. When experiments in the same study were performed using dead Jurkat cells that had been pre-labelled with the thymidine analogue bromodeoxyuridine (BrdU) prior to Adriamycin treatment, numerous BrdU-labelled fluorescent particles representing DNA were found to have accumulated in the nuclei of NIH3T3 cells. This indicated the involvement of cfChPs released from dying Jurkat cells in the aforementioned bystander effect. The phenomenon of chemotherapy-induced bystander effect is pictorially depicted in Fig. 1. During experiments conducted in vivo, in which chemotherapy-induced dying cancer cells were intravenously injected into mice, activation of H2AX and NF-κB was observed in the cells of the brain, liver, and lung in association with the integration of cfChPs released from the dying cells into the genomes of cells of these distant organs [14]. The bystander effects were eliminated following the concurrent use of three different cfChPs-deactivating agents: anti-histone antibody complexed nanoparticles (CNPs) [18], DNAse I, and a combination of the nutraceuticals R–Cu [14]. The latter findings incriminated cfChPs in the pathophysiology of the bystander effect in vivo.

Cell-free chromatin particles are also involved in the radiation-induced bystander effect. When NIH3T3 cells were treated with conditioned media obtained from irradiated cells, the upregulation of γH2AX, NF-κB, IL-6, and active caspase-3 were clearly observed [15]. There was also evidence of large-scale chromosomal aberrations including dicentric, acentric, fusion, and Robertsonian fusion chromosomes. The above bystander effects were abrogated when the conditioned medium was pre-treated with CNPs, DNase I, or R-Cu. In the same study, radiation treatment also led to systemic bystander effects; irradiation of the lower hemi-body of mice with γ-rays (10 Gy) led to the activation of H2AX, NFκB, IL-6, and active caspase-3 in brain cells [15]. Activation of these bystander biomarkers was abrogated by concurrent treatment with CNPs, DNase I, and R-Cu, thereby directly implicating cfChPs in systemic bystander effects [15].

### Activity and bystander effects of cfChPs

Cell-free chromatin particles released from chemotherapy-induced dying cells are apparently phagocytosed by bystander healthy cells. This was revealed by the micro-array analysis performed following co-culture of dying cells with healthy cells, which showed upregulation of pathways related to phagocytosis at 6 h [14]. The internalised cfChPs were found to integrate into the genomes of the host cells as confirmed by whole-genome sequencing, fluorescence in situ hybridisation (FISH) analysis, and the identification of many human Alu elements in the recipient cells [13, 14]. Genomic integration led to dsDNA breaks and activation of inflammatory and apoptotic pathways [13, 14] which are the critical elements of the bystander effect.

### Preclinical studies

**Fig. 2:**
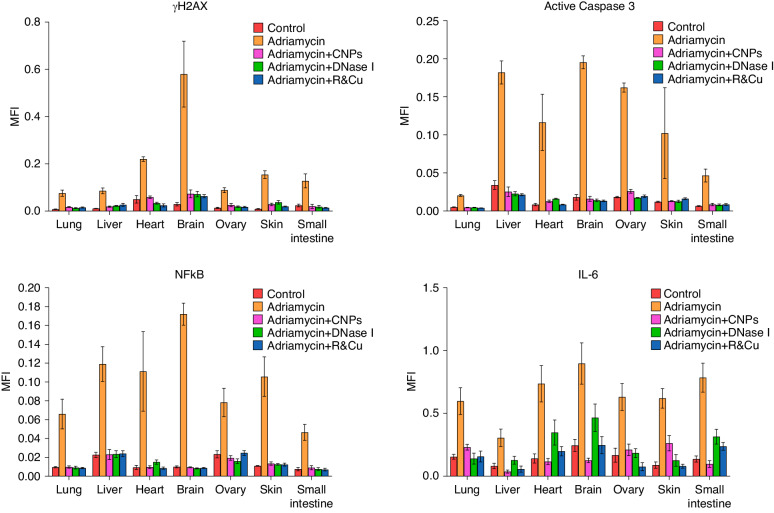
Prevention of cellular DNA damage, apoptosis, and inflammation in various organs by treatment with CNPs, DNase I, and R-Cu concurrently with a single i.p. injection of doxorubicin. The bars represent the mean values of five animals in each group. Statistical comparisons were carried out using unpaired Student’s *t*-tests. Mean ± SEM values of all groups were significantly lower than that of the doxorubicin group, with p values ranging between <0.01 and <0.0001 (reproduced with permission from [16] with modification).

Preclinical animal studies using the abovementioned cfChPs-deactivating agents have shown great promise [16]. Mice (5 per group) injected with the chemotherapeutic agent doxorubicin (Adriamycin) demonstrated a surge in the release of cfChPs from dying cells into the bloodstream, accompanied by an upsurge of inflammatory cytokines C-reactive protein, IL-6, TNF-α, and IFN-γ [16]. All values were highly significant when compared with untreated controls with *p* values ranging between <0.01 and <0.0001. Although the timing of these effects can vary, the most significant upregulation of NF-κB, IL-6, active caspase-3, and γ-H2AX is typically seen 18 h following chemotherapy. Blood collected following chemotherapy administration from mice treated with the three cfChPs-deactivating agents, viz. CNPs, DNAse I, and R-Cu led to significant downregulation of the inflammatory cytokines compared with untreated controls with *p* values ranging between >0.05 and >0.0001 [16]. When the Adriamycin-treated mice were dissected and their organs were examined for markers of DNA damage, apoptosis, and inflammation, a remarkable downregulation of the activated γH2AX, caspase-3, NF-κB, and IL-6 was observed with values that were almost identical to those in controls not treated with doxorubicin (*p* values ranging between >0.01 and >0.0001) [16]. These effects were observed in multiple organs, including the lungs, liver, heart, brain, ovaries, skin, and small intestine (Fig. 2). Identical results were observed in association with the administration of other chemotherapeutic agents, such as cyclophosphamide, cisplatin, methotrexate, and paclitaxel, with a significant downregulation of γH2AX and NF-κB in the brain and peripheral blood mononuclear cells following the use of cfChPs-deactivating agents. Furthermore, the occurrence of potentially life-threatening neutropenia was also significantly reduced with the concurrent use of neutralising agents [16]. Following the administration of a lethal dose of doxorubicin (20 mg/kg), the overall survival of mice (10 in each group) was also significantly prolonged using cfChPs-deactivating agents, with some mice surviving the lethal dose of doxorubicin with *p* values ranging between >0.05 and >0.0001 [16].

### The chemotoxicity enigma

**Fig. 3:**
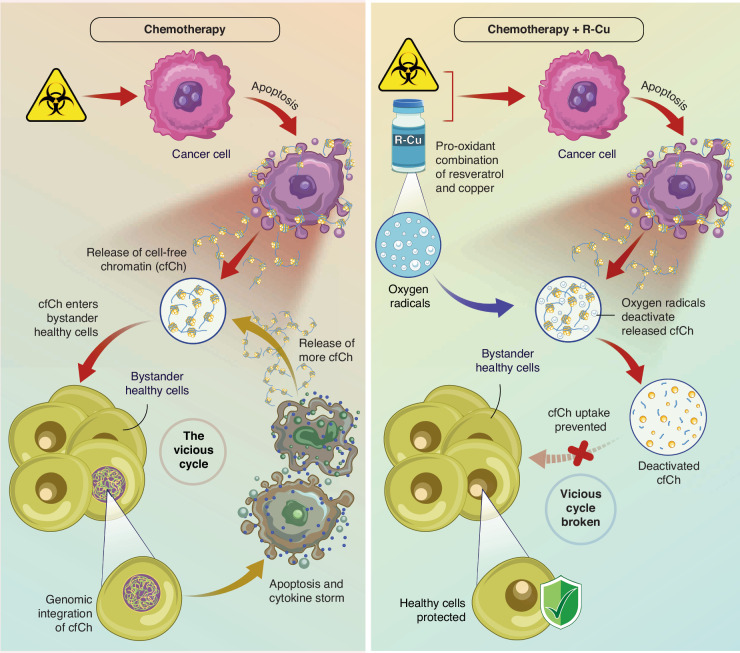
Schematic illustration of a vicious cycle of bystander cell death. Chemotherapy induced cell death sets in motion a vicious cycle of more cell death triggered by cfChPs released from dying cells. The vicious cycle can be broken by oxygen radicals generated by a combination of resveratrol with copper.

One particularly puzzling aspect of chemotoxicity is its chronology. Although the half-life of most chemotherapy drugs is approximately 24 h [19], their toxic side effects last for 2–3 weeks. For example, chemotherapy-induced bone marrow suppression reaches a nadir after 7–14 days, and the ensuing neutropenia often takes more than 3 weeks to recover. Similarly, chemotherapy-associated oral mucositis typically begins 5–7 days after initiating chemotherapy [20]. The results discussed above may help elucidate on the nature of this conundrum. Specifically, the aforementioned findings suggest that DNA damage, apoptosis, and inflammation directly resulting from the action of the chemotherapeutic agent(s) are minimal, and most toxic side effects are induced by cfChPs that are released from the initial round of drug-induced cell death. The latter mechanism sets in motion a cascading effect, whereby dying cells release more cfChPs, leading to a vicious cycle of further DNA damage, apoptosis, and inflammation in bystander cells, thereby amplifying and prolonging the toxic effects of chemotherapy. The vicious cycle of cascading bystander cell death resulting from chemotherapy treatment is illustrated in Fig. 3.

### Resveratrol–copper and its mechanism of action

**Fig. 4:**
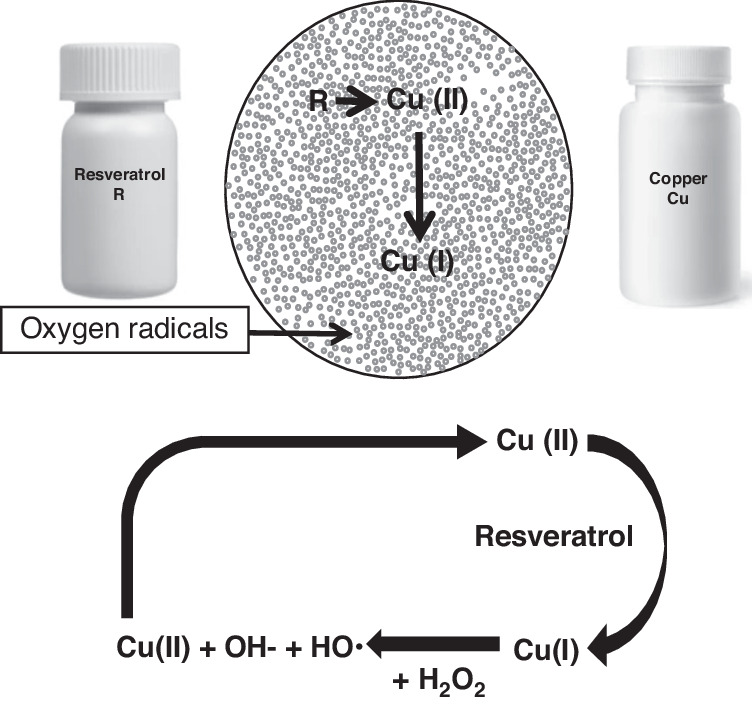
Admixing resveratrol and copper generates oxygen radicals via a Fenton-like reaction. Resveratrol catalyses the reduction of Cu(II) to Cu(I); the latter reacts with hydrogen peroxide (H_2_O_2_), leading to the formation of hydroxide ions (OH-), and a hydroxyl radical (•OH) and also regenerates Cu(II) in a cyclic manner. Reproduced with permission from [21] under creative commons license.

Of the three deactivating agents for cfChPs that have been previously utilised in preclinical studies, namely, CNPs, DNAse I, and R-Cu, the latter has been proven to be the agent of choice for therapeutic use in humans [21]. This is primarily because R-Cu can be easily administered orally and has demonstrated little toxicity in preclinical and clinical studies, even after prolonged administration [22]. Resveratrol (R) is a nutraceutical that has been extensively studied because of its antioxidant properties [23]. However, it acts as a prooxidant in the presence of copper (Cu), another widely researched nutraceutical [24]. R catalyses the reduction of Cu (II) to Cu (I), resulting in the generation of oxygen radicals via a Fenton-like reaction (Fig. 4) [25]. The oxygen radicals thus generated have previously been shown to be capable of cleaving plasmid pBR322 DNA [26, 27]. It was subsequently demonstrated that a combination of R and Cu can degrade genomic DNA and RNA [28] and can also deactivate cfChPs by degrading their DNA components [15, 16, 29]. Paradoxically, it has also been found that the DNA-degrading activity of R-Cu increases with a reduction in the molar concentration of Cu [28]. This finding has led to the establishment of the concentrations of R and Cu used in the clinical studies described below, wherein the molar ratio of R to Cu is maintained at 1:10^-^^4^.

### Clinical studies

Circulating cfChPs have been successfully isolated from the sera of patients with cancer and, when examined under an electron microscope, they exhibit a classical ‘beads-on-a-string’ appearance typical of nucleosomes (Supplementary Fig. 1) [13]. Utilising R-Cu as an essential deactivator of cfChPs formed the foundation of two phase II clinical trials [30, 31]. The first study was a dose-escalation pilot study in which 25 patients with multiple myeloma receiving hematopoietic stem cell transplantation were enrolled. Five patients formed the control group and the remaining 20 were administered one of two different doses of R-Cu (R = 5.6 mg and Cu = 560 ng, or R = 50 mg and Cu = 5 μg, both administered orally twice daily) 48 h before melphalan chemotherapy, continuing a twice-daily dose of R-Cu until 21 days post-transplantation [30]. The primary objective was to evaluate the immediate post-BMT toxicities, namely mucositis, nausea, vomiting, diarrhoea, the requirement of opioids, and the need for total parenteral nutrition. An objective evaluation of inflammation was performed by measuring the levels of inflammatory cytokines in the serum and saliva. The study found a statistically significant decrease in the incidence of grade 3 mucositis and the levels of inflammatory cytokines TNF-α and IL-1β. The incidence of grade III/IV oral mucositis was 100% in the control group versus 40% in the R-Cu groups (*p* = 0.039). Other adverse effects and cytokine levels showed non-significant reduction.

Another prospective open-label phase II single‑arm study was conducted to check for reduced haematological and non-haematological toxicities in 30 patients with advanced gastric cancer following docetaxel-based multi-agent chemotherapy [31]. Patients received R-Cu (dosage: R = 5.6 mg and Cu = 560 ng) orally thrice daily concurrently with chemotherapy for 6 months or until evidence of disease progression, whichever occurred sooner. Although the cumulative incidence of overall and haematological toxicity declined, this failed to reach statistical significance. However, the incidence of more troublesome non-haematological toxicities, such as hand-foot syndrome, diarrhoea, and vomiting, was dramatically reduced in the R-Cu group compared to a historical cohort.

### Effect of R-Cu on tumour cells

In addition to the role of R-Cu in minimising chemotoxicity, it may have potential therapeutic effects on tumour cells. The oxygen radicals that are generated by the R-Cu combination not only deactivate extracellular cfChPs to prevent the damaging bystander effects of chemotherapy, but they can also enter into the tumour to deactivate intratumoural cfChPs that are derived from dying cancer cells. A recent study in patients with advanced squamous cell carcinoma of the oral cavity (OSCC) detected the copious presence of cfChPs in the tumour microenvironment (TME) derived from dying cancer cells, which were eradicated following 2 weeks of twice-daily oral administration of R-Cu [32]. Eradication of cfChPs from TME was accompanied by marked downregulation of 23 biomarkers representing 10 hallmarks of cancer, including 5 immune checkpoints, defined by Hanahan and Weinberg [33] with *p* values ranging between >0.063 and >0.000. This study suggested that cfChPs from dying cancer cells are global instigators of cancer hallmarks in surviving cancer cells and raised the hypothesis that prolonged treatment with R-Cu may lead to the reversal of the malignant phenotype of cancer [32].

## Conclusion

Oncological treatments can cause a range of adverse effects associated with morbidity and mortality independent of the underlying cancer. Advances in our knowledge of the pathophysiological mechanisms underlying chemotoxicity are essential to facilitate the development of effective treatments that improve the outcomes and quality of life of patients with cancer. Collectively, the laboratory, animal, and clinical studies described in this article corroborate the hypothesis implicating cfChPs in “bystander effect” chemotoxicity, as well as the role of R-Cu as a potent cfChPs-deactivating agent. Larger clinical studies with greater statistical power may further clarify the reduction in the incidence of various toxicities associated with the use of R-Cu and facilitate our understanding of additional pathophysiological mechanisms. In addition, a greater focus on studying the cellular response to cfChPs may reveal novel mechanisms of toxicity associated with oncological treatments and further our understanding of the management of the adverse effects of chemotherapy.

## Supplementary information

Supplementary Figure

## Data Availability

All data supporting the findings of this study are available within the article and the cited studies.
