# Supplementary material for: New light on chemotherapy toxicity and its prevention
Source: BJC Rep. 2024 May 22;2:41. doi: 10.1038/s44276-024-00064-8 (PMC11524128; doi:10.1038/s44276-024-00064-8)
Supplement: Supplementary file 1 — Supplementary Figure [file 44276_2024_64_MOESM1_ESM.docx]

**Supplementary Figure 1**


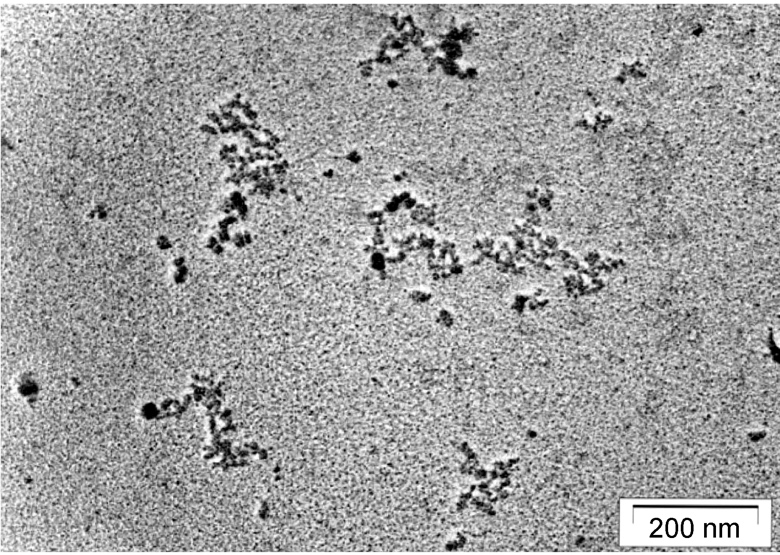


**Supplementary Figure 1:** Representative electron microscopy image of cfChPs isolated from the sera of patients with cancer showing a “beads-on-a-string” appearance typical of chromatin. Reproduced with permission from ref. 13.
